# Metabolic Conservation and Diversification of *Metarhizium* Species Correlate with Fungal Host-Specificity

**DOI:** 10.3389/fmicb.2016.02020

**Published:** 2016-12-16

**Authors:** Yong-Jiang Xu, Feifei Luo, Bing Li, Yanfang Shang, Chengshu Wang

**Affiliations:** CAS Key Laboratory of Insect Developmental and Evolutionary Biology, Institute of Plant Physiology and Ecology, Shanghai Institutes for Biological Sciences, Chinese Academy of SciencesShanghai, China

**Keywords:** *Metarhizium*, secondary metabolism, gene clusters, divergent evolution, metabolomics, insect host adaptation

## Abstract

The ascomycete genus *Metarhizium* contains several species of insect pathogenic fungi ranging from specialists with narrow host ranges to generalists that can infect diverse invertebrates. Genetic and metabolic conservations and diversifications of *Metarhizium* species are not well understood. In this study, using the genome information of seven *Metarhizium* species, we performed a comparative analysis of gene clusters involved in secondary metabolisms (SMs) in these species. The results revealed that the generalist species contain more SM gene clusters than the specialists, and that both conserved and divergent evolutions may have occurred in SM genes during fungal speciation. In particular, the loss/gain events, as well as gene mutagenesis, are evident for the gene cluster responsible for the biosynthesis of non-ribosomal cyclopeptide destruxins. The presence of conserved SM gene clusters in *Metarhizium* and other divergently evolved insect pathogenic fungi implies their link to fungal entomopathogenicity. Mass spectrometry based metabolomic analyses were also conducted to investigate the chemical diversities of seven *Metarhizium* species. Consistent with the evolutionary relationships of SM genes among the seven species, significant differences are observed in fungal metabolic profiles, whether the same or different metabolites are produced in different species. Clustering analysis based on the metabolome data revealed that *Metarhizium* species could be grouped based on their association to fungal host specificity. Our metabolomics-based methods also facilitate the identification of bioactive metabolites that have not been reported previously in *Metarhizium*. The results of this study will benefit future investigations of the chemical biology of insect-fungal interactions.

## Introduction

A number of species have been identified in the ascomycete *Metarhizium* genus that are insect pathogenic fungi (Kepler et al., 2014). Among these, *Metarhizium anisopliae* and *M. acridum* have been developed as biocontrol agents to mitigate insect pest populations (de Faria and Wraight, 2007; St Leger and Wang, 2010). Of particular interest is the divergence of *Metarhizium* species to adapt to different varieties of invertebrate hosts. For example, in contrast to the invasion of versatile hosts by the generalist species such as *M. anisopliae* and *M. robertsii*, *M. acridum*, and *M. album* are more specific toward orthopteran and hemipteran insect hosts, respectively (Wang et al., 2012). Pan-genomic analyses of *Metarhizium* species revealed the expansion/contraction of protein families associated with fungal host adaptation (Hu et al., 2014). Transcriptional and developmental differences have been also observed when *Metarhizium* species were challenged with host or non-host cuticles. These differences include the up-regulation of divergent G-protein coupled-receptors, differential expression of down-stream singling pathway genes, and finally, production of the infection structure appressoria to determine fungal host recognition and infection (Wang and St Leger, 2005; Gao et al., 2011; Chen et al., 2016). Despite these advances, metabolomic and physiological differences among the *Metarhizium* species are largely unclear when the fungi are grown under the same or different conditions.

Different bioactive metabolites have been isolated from *Metarhizium* species, including the well-known non-ribosomal peptide destruxins (Dtxs) (Molnar et al., 2010; Gibson et al., 2014), which are non-selectively insecticidal. Interestingly, the toxin-producing gene cluster was not present in the genomes of the specialist species *M. acridum* and *M. album* (Wang et al., 2012). Presence or absence of gene clusters involved in secondary metabolisms (SMs) has been frequently observed in the genomes of closely related or unrelated fungal species. Horizontal transfer of gene clusters between species has been suggested (Khaldi et al., 2008; Slot and Rokas, 2011). Divergent evolution also contributed to the diversity of fungal SMs. For example, a highly conserved polyketide synthase (PKS) involved in the biosynthesis of dihydroxynaphthalene (DHN) melanin is present in the genomes of *Metarhizium* species. However, DHN-type melanin could not be produced by *Metarhizium* due to the divergent evolution of the tailoring enzyme laccase (Chen et al., 2015). On the other hand, differential regulation (including epigenetic control) of secondary metabolic gene expression largely determines chemical diversity in fungi (Yu and Keller, 2005; Keller, 2015). Relative to the number of identified gene clusters in each species (Hu et al., 2014), chemical diversity and the associated genetics in different *Metarhizium* species are still poorly understood (Donzelli and Krasnoff, 2016).

High-resolution technique oriented metabolomics can systematically identify and quantify metabolites from a biological sample, and has emerged as a powerful tool for microbiological studies (Oldiges et al., 2007; Xu et al., 2014). For example, metabolomic analyses of the insect pathogens *M. brunneum* and *Beauveria bassiana* cultured in different conditions revealed that both fungi produce significantly different arrays of secondary metabolites during infectious and saprophytic growths (de Bekker et al., 2013). Similarly, the chemical diversity of *B. bassiana* varied in association with the host and non-host growth cultures, and oxidative stress conditions (Luo et al., 2015; Xu et al., 2015; Zhang et al., 2016). The exploration of metabolomic technique could also help identify known metabolites that have not been reported in a given sample or organism. For example, metabolomic analysis of *B. bassiana* mycelial samples identified the macrocyclic lactone, doramectin and a magnesium-containing antibiotic, magnesidin that were originally identified in bacteria (Luo et al., 2015). Our previous analysis of metabolic dynamics in silkworm (*Bombyx mori*) larvae identified a mulberry leaf component maclurin in insect hemolymph that reduced the feeding tendency of insects infected with *B. bassiana*, thus indicating an anti-feeding effect on insects infected with fungal pathogen (Xu et al., 2015). Metabolomic analysis has been also used to investigate and compare the intracellular metabolic responses among different yeast species (Christen and Sauer, 2011).

In this study, we performed a comparative analysis of gene clusters involved in SMs in seven *Metarhizium* species. Metabolomic analyses of seven species grown in the same medium were also conducted by using gas chromatography (GC) and liquid chromatography-mass spectrometry (LC-MS). Consistent with the conserved and divergent evolution of SM gene clusters, our metabolome data indicate that fungal metabolic profiles are linked to fungal host specificity. In addition, the high resolution of the MS technique facilitated the identification of a few compounds that have not been reported previously in *Metarhizium* species.

## Materials and Methods

### Fungal Strain and Culture Conditions

Seven strains of genome-sequenced *Metarhizium* species were used in this study (Hu et al., 2014), including *M. album* (species abbreviated MAM, strain ARSEF 1941), *M. acridum* (MAC, CQMa 102), *M. majus* (MAJ, ARSEF 297), *M. guizhouense* (MGU, ARSEF 977), *M. brunneum* (MBR, ARSEF 3297), *M. anisopliae* (MAN, ARSEF 549), and *M. robertsii* (MAA, ARSEF 23). Fungal cultures were maintained on potato dextrose agar (PDA, BD Difco) at 25°C for 2 weeks, and the spores were harvested to prepare spore suspensions (10^6^ conidia/ml). For liquid culture incubation, an aliquot (100 μl) of the spore suspension from each species was inoculated into a 100 ml flask containing 25 ml of Sabouraud dextrose broth (SDB, BD Difco) for 7 days at 26°C and 120 rpm on a rotating shaker. Five replicates were maintained for each species.

### Bioinformatics Analysis of Secondary Metabolic Gene Clusters

Based on our previously obtained genome information of seven *Metarhizium* species (Gao et al., 2011; Hu et al., 2014), whole-genome bioinformatic re-analyses of SM gene clusters were performed for each species using the updated version of antiSMASH (ver. 3.0.4) (Weber et al., 2015). Blast comparison to other known gene clusters was conducted using the in-house algorithm of fast and sensitive protein alignment method, and the clusters are ranked based on the cumulative BlastP bit scores between the gene clusters. Representative gene clusters conserved in the seven species were selected and illustrated for comparison.

### Sample Preparation

Mycelia harvested from the SDB cultures were washed three times with ice-cold sterile water, followed by freezing in liquid nitrogen and lyophilization. An aliquot of the mycelial powder (2 mg each) was treated with 200 μl ice-cold methanol spiked with 10 μg/ml of N-(9-fluorenylmethoxycarbonyl)-glycine (Fmoc-glycine, J&K Scientific Company, Shanghai, China) as an internal standard. The samples were centrifuged at a maximum speed for 10 min at 4°C, and the supernatants (75 μl each) were directly used for LC-MS analysis. Aliquots (75 μl each) of the supernatants were dried under nitrogen, derived using 200 μL methoxyamine (50 μg/ml in pyridine, 37°C for 16 h) and followed by the addition of 200 μL of *N*-methyl-*N*-trimethylsilyltrifluoroacetamide (J&K Scientific Company, Shanghai, China) at 37°C for 2 h. After centrifugation, the derived sample (1.0 μl) was injected splitlessly into a gas chromatography-mass spectrometry (GC-MS) system for analysis (Xu et al., 2015).

### High Performance Liquid Chromatography (HPLC), GC-MS, and LC-MS Analyses

The extracted samples were first analyzed using a LC-20AD HPLC system (Shimadzu Scientific Instruments, Japan) equipped with a C18 reverse phase column (Athena C18; particle size: 5 μm; length: 4.6 mm × 250 mm) and an SPD-M20A UV detector. For the detection of destruxins, the gradient elution of deionized water and methanol was set from 90:10 to 10:90 for 40 min at a flow rate of 1 mL/min, which was monitored at a wavelength of 215 nm (Wang et al., 2003). For GC-MS analysis, the derived samples (1.0 μl each) were individually injected splitlessly into a Thermo DSQ GC/MS system (Thermo Fisher Scientific, Waltham, MA, USA) equipped with a fused-silica capillary column HP-5MSI (30 m × 0.25 mm inner diameter, 0.25 μm film thickness) using a Thermo 1310 Series Autosampler. The inlet temperature was set at 250°C. Helium was used as the carrier gas with a constant flow rate at 1 ml/min through the column. The initial oven temperature was set at 70°C for 1 min, and then increased to 250°C at a rate of 10°C/min and further increased to 300°C at a rate of 25°C/min for 6 min. The transfer line temperature was set at 280°C and the ion source temperature set at 230°C. The mass spectrometer was operated in an electron impact mode (70 eV). Data acquisition was performed using a full scanning mode from m/z 50–550.

Liquid chromatography-mass spectrometry analysis was performed using an Agilent 1100 HPLC system (Waldbronn, Germany) equipped with a Quadrupole Time-of-Flight (Q-TOF) mass detector controlled by a MassHunter workstation. The column used for separation was an Inertsil ODS-3 (4.6 mm × 250 mm, 5 μm) (GL sciences, Japan). The oven temperature was set at 40°C, and the column was eluted using a mobile phase consisting of 0.1% formic acid in water (A) and 0.1% formic acid in methanol (B). After sample loading (10 μl each), the initial elution condition was set at 5% of B, and followed by the solvent gradients: from 5% B to 100% B within 40 min, then hold for 5 min at a flow rate of 1 ml/min. The ESI-MS were acquired in both positive and negative ion modes. The ion spray voltage was set at 4,000 V. The heated capillary temperature was maintained at 350°C. The drying gas and nebulizer nitrogen gas flow rates were set at 12 l/min and 30 psi. For full scan mode analysis, spectra were stored from *m/z* 50 to 1000 (Xu et al., 2015).

### Identification and Characterization of Metabolites

For GC-MS analytic data, a mass spectral library (NIST 11, Thermo Fisher Scientific) was used to identify metabolites based on the retention time (RT) index and mass-spectral similarity match (more than 85%). For LC-MS data, the features were temporarily identified based on accurate mass through matching databases: Combined Chemical Dictionary (version 8.1), Massbank, PubChem or METLIN. Furthermore, MS/MS information (fragment pattern) from different collision energies (low, medium, and high) was used to confirm the identified metabolites (Xu et al., 2015).

### Data Processing

Each chromatogram obtained from GC-MS or LC-MS analysis was processed for baseline correction and area calculation using the software MZmine (ver. 2.0). The data were combined into a single matrix by aligning peaks with the same m/z and RT for GC-MS and LC-MS data, respectively. The area of each peak was normalized to the internal standard (Fmoc-glycine) in each data set. The data were subsequently filtered by the presence of peaks in at least 80% of each sample category. The missing values were replaced with a half of the minimum value obtained in the data set for processing without divide-by-zero problems (Xia et al., 2009; Xu et al., 2015). The pre-processed GC-MS and LC-MS data were exported to the program SIMCA-P (ver. 11.0, Umetrics AB, Umeå, Sweden) for analysis and visualization by multivariate statistical methods. Principal component analysis (PCA) was used to find the optimal separation of clusters. Heat maps of identified metabolites were generated using the program MultiExperiment Viewer (ver. 4.8).

## Results

### Evolutionary Convergence and Divergence of Secondary Metabolisms

**FIGURE 1:**
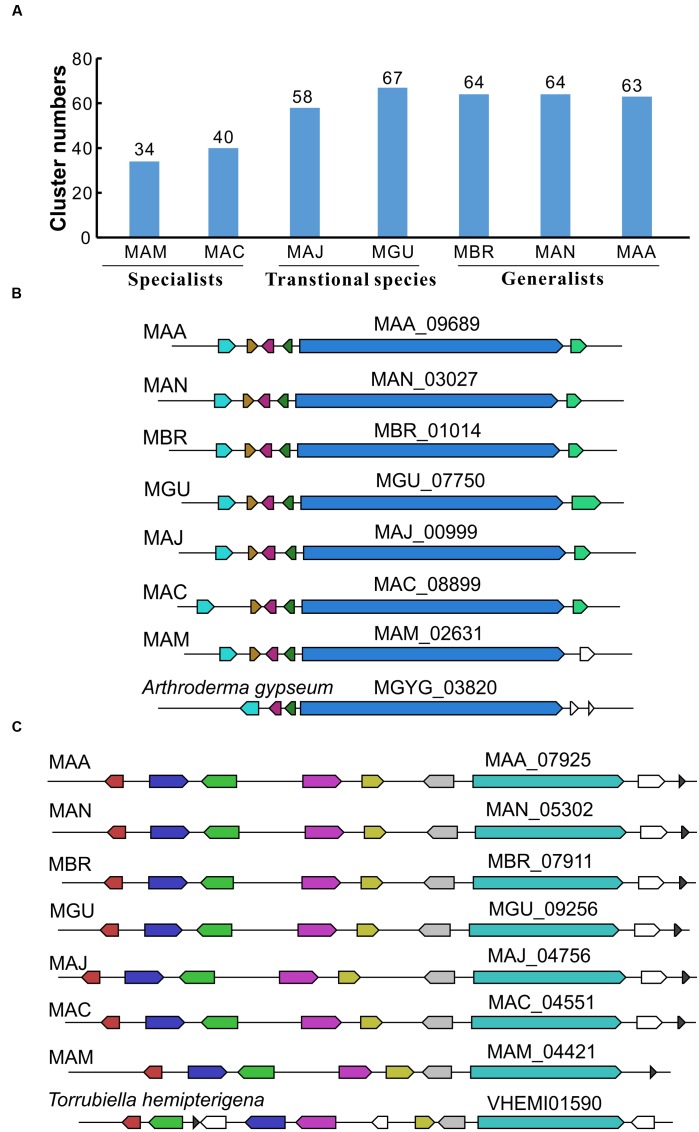
**Secondary metabolic gene clusters identified in seven *Metarhizium* species.** **(A)** Number of secondary metabolic gene clusters encoded in the genome of each *Metarhizium* species. **(B)** A representative highly conserved NRPS gene cluster present in seven *Metarhizium* species and the insect pathogen *Torrubiella hemipterigena*. **(C)** A representative highly conserved PKS gene cluster present in seven *Metarhizium* species and *T. hemipterigena*. Core gene numbers are shown above each gene cluster, and the genes labeled in the same color show synteny with each other. Abbreviated *Metarhizium* species are MAM for *M. album*; MAC, *M. acridum*; MAJ, *M. majus*; MGU, *M. guizhouense*; MBR, *M. brunneum*; MAN, *M. anisopliae*; MAA, *M. robertsii*.

Gene cluster re-analysis indicated that the genomes of the generalist species such as *M. robertsii* and *M. anisopliae* encode more SM gene clusters (63.7 on average) than the specialist species *M. acridum* and *M. album* (37 on average). The transitional species, *M. majus* and *M. guizhouense* with intermediate insect host ranges also have higher numbers of gene clusters (62.5 on average) than the specialists (**Figure 1A**). By using the basal species *M. album* (with the fewest number of 34 SM clusters) as a reference, blast analyses of the clusters identified were conducted to investigate the evolution/conservation of SM gene clusters among seven *Metarhizium* species. The results indicated that there are 12 clusters conserved in the genomes of the seven species. For example, a non-ribosomal peptide synthetase (NRPS) gene cluster is present not only in the seven *Metarhizium* species but also in the dermatophyte *Arthroderma gypseum* (**Figure 1B**). Likewise, a PKS gene cluster is highly conserved in the seven *Metarhizium* species and *Torrubiella hemipterigena* (**Figure 1C**). However, we also identified eight species-specific SM gene clusters present only in *M. album*, and five clusters present only in two *Metarhizium* species. In particular, a NRPS (MAM_01762 vs. MAC_07604) and a PKS (MAM_07310 vs. MAC_00177) gene clusters are conserved only in two specialists *M. album* and *M. acridum*. We also found the gene clusters present only in the generalist species, e.g., a PKS cluster present only in *M. robertsii* (MAA_08283), *M. anisopliae* (MAN_09465), and *M. brunneum* (MBR_10208).

**FIGURE 2:**
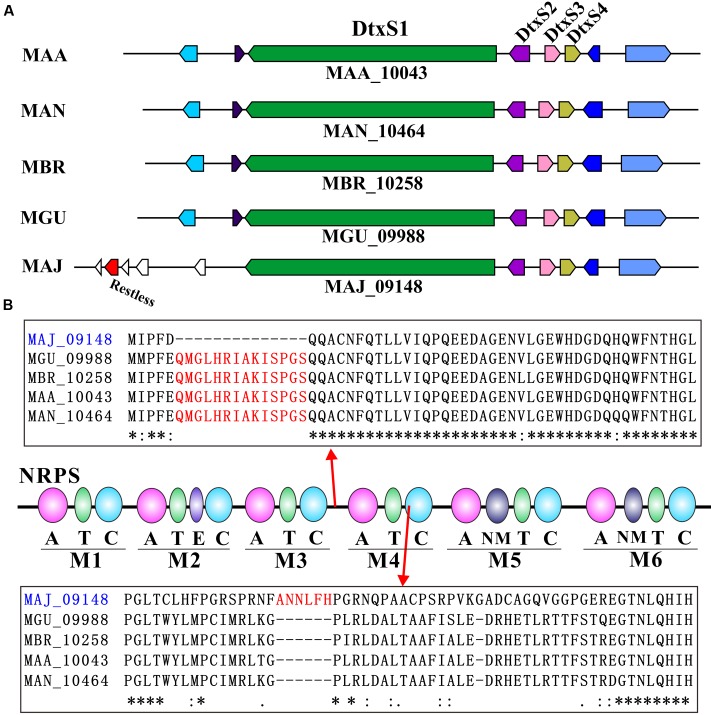
**Destruxin biosynthesis gene cluster and NRPS sequence analyses.** **(A)** Analysis of the destruxin biosynthetic gene cluster in five *Metarhizium* species. **(B)** Sequence comparison of NRPS DtxS1 in five *Metarhizium* species. Asterisks represent the consensus amino acid residues among different proteins.

It has been demonstrated that different *Metarhizium* species have the abilities to produce the insecticidal cyclopeptides, destruxins (Amiri-Besheli et al., 2000; Wang et al., 2012). Our analysis of the seven *Metarhizium* genome data indicated that the toxin-producing gene clusters are present in five non-specialist species, however, the upstream region of the cluster is divergent in *M. majus* (**Figure 2A**). In particular, a transposon-like gene (MAJ_09144) encoding a hAT family of Restless-like transposase, is adjacent to the cluster. Further analysis and comparison of the biosynthetic protein sequences revealed that, relative to other orthologs, the DtxS1-like NRPS in *M. majus* (MAJ_09148) contains an indel with the deletion of 14 amino acids between the modules 3 and 4, and insertion of six amino acids in the condensation domain of module 4 (**Figure 2B**). Thus, the above data suggest that both conserved and divergent evolutions may have occurred in different *Metarhizium* species for the production of bioactive secondary metabolites.

### Multivariate Data Analyses

**FIGURE 3:**
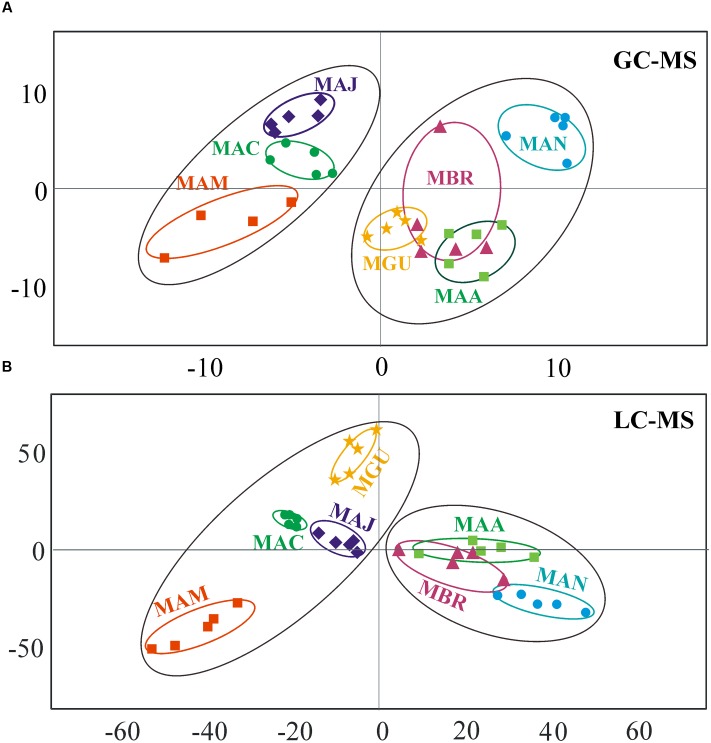
**Statistical analysis of metabolomic profiles in seven *Metarhizium* species.** **(A)** PCA plotting based on the data obtained from GC-MS analysis. **(B)** PCA plotting based on the data obtained from LC-MS analysis.

Metabolomics analysis was performed using the mycelia harvested from seven *Metarhizium* species cultured in SDB for a week. For GC-MS data, a three components model revealed that 98.1% of the variance could be used for PCA analysis. We used the first two components for analysis and found that the seven *Metarhizium* species clustered into two well-separated groups with a R2X of 0.516 and a Q2 value of 0.754 (**Figure 3A**). One group with a positive first component contained the generalist species *M. robertsii*, *M. anisopliae*, and *M. brunneum* as well as the transitional species *M. guizhouense*. The other group with a negative first component contained the specialist species *M. album* and *M. acridum* as well as the transitional species *M. majus*. Based on the LC-MS data, PCA analysis also resulted in two well-separated groups with a robust modeling fit: R2X value of 0.881 and a Q2 value of 0.725. The two clusters were associated again with fungal host ranges, i.e., the generalist species were assigned to one group while the specialists and transitional species with narrow host ranges were in the second group (**Figure 3B**).

### Identification of Metabolites Produced in *Metarhizium* Species

**FIGURE 4:**
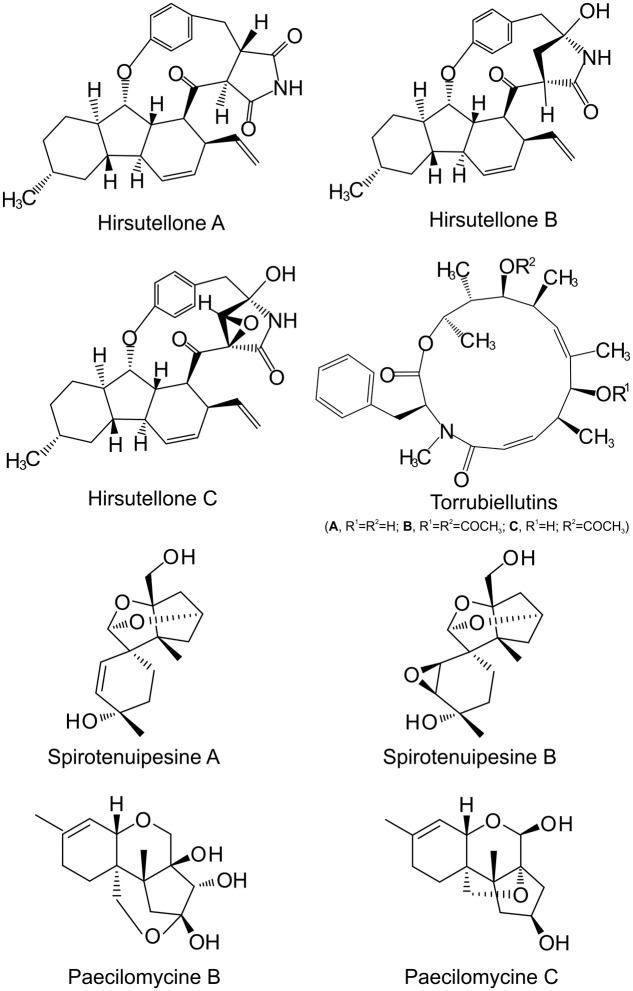
**The structures of representative compounds identified from *Metarhizium* species**.

Based on the obtained GC-MS data, 34 metabolites from different *Metarhizium* species were identified as analogs to known molecules, including amino acids, organic acids, carbohydrates, and sterols (**Supplementary Table S1**). Not surprisingly, these components could be identified in all *Metarhizium* species, although at varied levels, due to their importance in fungal physiology and biology. For example, the identified glycerate-3-phosphate is an essential component in the glycerol phosphate pathway that leads to the biogenesis of lipid droplets and maintenance of phospholipid homeostasis that have been functionally verified in *M. robertsii* (Gao et al., 2013, 2016). In LC-MS analysis, we identified 102 compounds with known structures based on the database searches. These include destruxins, alkaloids hirsutellones (A–C), macrocyle torrubiellutins (A–C), naphthoquinones naphthgeranines (B–D), and trichothecanes spirotenuipesines (A, B), paecilomyces (B, C), and tenuipesine (A) (**Figure 4**; **Supplementary Table S2**). These alkaloids and trichothecanes have not been reported previously in *Metarhizium* species.

**FIGURE 5:**
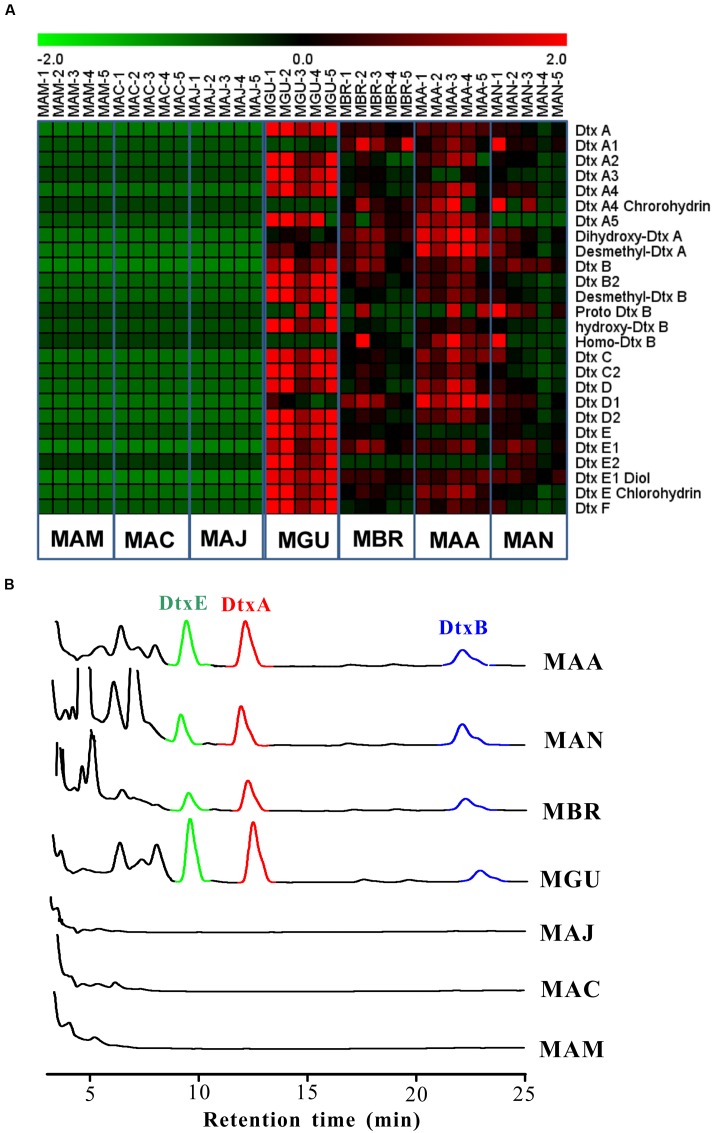
**Liquid chromatography-mass spectrometry (LC-MS) and chromatographic analyses of destruxin analogs production or non-production in seven *Metarhizium* species.** **(A)** Heat map analysis of the production of destruxin analogs in the mycelia of *Metarhizium* species. **(B)** HPLC analysis of destruxin production in the culture filtrates of different *Metarhizium* species. The spores of *Metarhizium* species were inoculated in the SDB medium for 10 days, and the culture filtrates were used for HPLC analysis.

**FIGURE 6:**
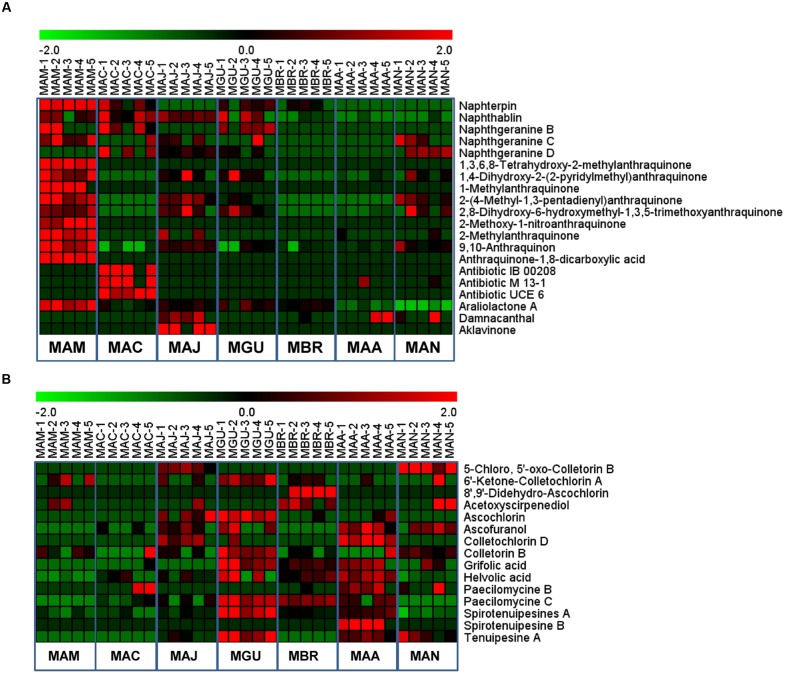
**Heat map analysis of selected polyketones** **(A)** and terpenoids **(B)** produced in different *Metarhizium* species.

Unlike the constitutive presence of the primary compounds in all examined *Metarhizium* species (**Supplementary Table S1**), the secondary metabolites fell into two different categories; some were produced by all examined species while others were asymmetrically present in different *Metarhizium* species samples (**Supplementary Table S2**; **Figures 5** and **6**). For example, destruxins were not detected in *M. album*, *M. acridum*, and *M. majus* (**Figure 5A**). Considering that the Dtx-producing gene cluster is present in the genome of *M. majus* (**Figure 2A**), we further performed a HPLC analysis of fungal culture filtrates. Consistent with our previous finding (Wang et al., 2012), the results confirmed that *M. majus* with a mutated *DtxS1*-like gene (**Figure 2B**) and those specialists without the toxin-producing gene cluster could not produce destruxins (**Figure 5B**). Interestingly, tenuipesine A was only detected in the Dtx-producing species (**Supplementary Table S2**). However, we also found that 16 out of 120 known metabolites could be detected in all *Metarhizium* species. For example, the antibacterial nortriterpenoid helvolic acid was detected in all seven *Metarhizium* species, which was previously reported in *M. anisopliae* (Lee et al., 2008). In addition, hirsutellones A-C, torrubiellutin C, and spirotenuipesine A were also detected in all seven species of *Metarhizium* (**Supplementary Table S2**).

### Relative Quantification of Metabolites Produced in *Metarhizium* Species

We also quantified 61 selected secondary metabolites produced in different *Metarhizium* species based on the Selected Ion Monitoring mode of data acquisition in LC-MS. The results indicated that the level of Dtx varied significantly among the toxin-producing species. The highest amounts were produced by *M. guizhouense* followed by *M. robertsii*, *M. brunneum*, and *M. anisopliae* (**Figure 5A**); specifically, the levels of Dtx E and Dtx A in *M. guizhouense* were >2-fold higher than in *M. brunneum* and *M. anisopliae* (**Supplementary Table S2**). Interestingly, the specialist *M. album* produced higher amounts of polyketides than the other species (**Figure 6A**). The accumulated level of terpenoids was relatively higher in *M. guizhouense*, *M. robertsii*, and *M. brunneum*, e.g., the spirotenuipesine A levels were >2-fold higher in *M. guizhouense* than in the other species (**Figure 6B**; **Supplementary Table S2**). Thus, besides the pathway-specific alterations in regulation, genetic variation of global regulators among different species may potentially lead to the difference in the amount of metabolite productions.

## Discussion

Genomic analyses indicated that entomopathogenic fungi belonging to the *Metarhizium* genus have the potential to synthesize a plethora of secondary metabolites (Hu et al., 2014). However, apart from the handful of small molecules previously identified in *Metarhizium*, the capacity of secondary metabolite production in *Metarhizium* species has not been fully investigated (Donzelli and Krasnoff, 2016). In this study, re-analysis of the genome data revealed the occurrence of both conserved and divergent evolution of SM gene clusters among the seven *Metarhizium* species analyzed. In particular, the gene cluster involved in the production of insecticidal destruxins is found to have evolved divergently in the different species, including the incident of lineage/species-specific mutagenesis in the toxin-biosynthesis gene that likely disabled the production of toxins. By employing integrated metabolomic approaches, we found that the obtained data allowed the grouping of *Metarhizium* species into clusters correlating with fungal host ranges. In addition, we found that the pattern of small molecule production is generally consistent with the conserved and divergent evolution of SM gene clusters.

Secondary metabolic conservation and diversification have been frequently observed in fungi. It includes two different scenarios: closely related fungi containing divergently evolved SM gene clusters that produce structurally different small molecules, whereas divergently evolved fungi producing the same metabolites (Keller, 2015). Consistent with this notion, the analysis of seven *Metarhizium* species in this study demonstrated clearly the conserved and divergent evolution of SM genes. Among > 30 SM gene clusters encoded by different species, less than half (12 clusters) are conserved in all examined *Metarhizium* species while the remaining clusters are either lineage- or species-specific. In particular, the gene cluster responsible for Dtx production is not present in the host-specific fungi *M. album* and *M. acridum*. Considering that destruxins are non-selectively insecticidal (Pedras et al., 2002; Gibson et al., 2014), the absence of this gene-cluster in host-specific fungi but its presence in non-specific *Metarhizium* species shows a clear association with the evolution of fungal host specificity. On the other hand, this observation also raises the question whether the gene cluster was lost in specialists or acquired in non-specialist species. *Metarhizium* species evolved after the divergence of the ascomycete plant pathogenic fungi (Shang et al., 2016; Wang et al., 2016), and the specialist species evolved first (Hu et al., 2014). To adapt to diverse insect hosts, it is possible that the non-specialists acquired the toxin-producing gene cluster during their speciation processes. However, considering that Dtx B can be produced by plant pathogens (Wang et al., 2012), it cannot be ruled out that the ancestor of *Metarhizium* contained the gene cluster but the specialist species lost it. Gain or loss event could also be applied to explain the presence/absence of other lineage/species-specific clusters. For example, comparative analysis of the putative genes involved in helvolic acid biosynthesis indicated the absence of the cluster in the genomes of the specialists *M. acridum* and *M. album* (Donzelli and Krasnoff, 2016). Besides the incidence of the horizontal transfer of gene clusters (Khaldi et al., 2008), the driving force(s) leading to the gain/loss evolution of SM genes among those closely related fungal species remains elusive.

Based on our metabolomic data, PCA analysis divided the examined *Metarhizium* species into separate groups in association with fungal host ranges (**Figure 3**). In particular, the small molecule profiles clearly distinguished between the generalist and non-generalist species (**Figure 3B**). These findings are consistent with previous studies that demonstrated the contribution of secondary metabolites to fungal virulence; e.g., destruxins in *Metarhizium* (Wang et al., 2012), and beauvericin and oosporein in *B. bassiana* (Xu et al., 2008; Feng et al., 2015). For destruxins, about 40 analogs have been identified (Pedras et al., 2002). Based on chromatography, however, only a few common destruxin derivatives such as Dtx A, Dtx B, and Dtx E could be detected in any given *Metarhizium* species or strain (Amiri-Besheli et al., 2000; Wang et al., 2004). In this study, the metabolomics analysis based on the highly sensitive LC-MS technique indicated that additional derivatives could be produced by fungi grown in an artificial medium (**Figure 5A**). This supports previous results that the adenylation of NRPS DtxS1 domain 3 can select either proline (for Dtx A-F series) or pipecolic acid (for Dtx A_1_-F_1_ series) as substrate, and the DtxS1 domain 4 can use either isoleucine (for Dtx A-F series) or valine (for Dtx A_2_-F_2_ series) (Donzelli et al., 2012; Wang et al., 2012). It is unexpected to find that the specialist *M. album*, with the fewest number of PKSs, produced higher amounts and varieties of polyketide molecules compared to other species (**Figure 6A**). Future studies are required to determine the mechanism(s) involved in the differential control of SMs in *Metarhizium* species.

Our metabolomics analysis also identified small molecules that were not previously reported in any *Metarhizium* species (**Figure 4**; **Supplementary Table S2**). Interestingly, these metabolites were originally identified from other insect pathogenic fungi except the *Streptomyces*-origin of polyketones naphthgeranines (Wessels et al., 1991). For example, torrubiellutins were identified from the insect pathogen *T. luteorostrata* (Pittayakhajonwut et al., 2009), hirsutellones from *Hirsutella nivea* (Isaka et al., 2005), and trichothecanes tenuipesine (Kikuchi et al., 2004a), spirotenuipesines (Kikuchi et al., 2004b), and paecilomycines (Kikuchi et al., 2004c) from the insect pathogen *Paecilomyces tenuipes* (now re-classified as *Isaria tenuipes*). The identification of similarly conserved SM gene clusters among the *Metarhizium* and *Torrubiella* species (**Figure 1C**) supports, at least in part, the production of same metabolites by different fungal species that has been frequently observed (Gibson et al., 2014). This finding would suggest that these metabolites may contribute to fungal entomopathogenicity. Likewise, by using a high resolution MS technique, the immunosuppressive compounds α-pyrone diterpenoids subglutinols were identified in *M. robertsii* (Kato et al., 2016). In contrast to the compounds mentioned above, these diterpenoids were originally identified from the plant pathogen *Fusarium subglutinans* (Lee et al., 1995). Future studies are required not only to verify the production of these molecules in *Metarhizium* species but also to elucidate their biosynthetic mechanisms and biological functions.

There are also the cases that the known products identified from *Metarhizium* species have not been detected in our metabolome analysis. These compounds include the non-ribosomal cyclopeptides ferricrocin, serinocyclins, and metachelins; the polyketides aurovertins; the polyketide/peptide hybrids metacridamides, NG-391 and NG-393; the terpenoids viridoxins, metarhizins and ovalicins; and the indolizidine alkaloids swainsonine and fungerins (Gibson et al., 2014; Donzelli and Krasnoff, 2016). First, it could be the reason of species-specific production of certain metabolites, which can be supported by the presence of species/lineage-specific gene clusters in different *Metarhizium* species. For example, the diterpenes viridoxins and metarhizins were identified from *M. flavoviride* (Gupta et al., 1993; Kikuchi et al., 2009). These diterpenoids have not yet been reported in other *Metarhizium* species. Second, the growth media may determine fungal chemical diversity. For example, aurovertins F-H were identified from *M. anisopliae* grown in a culture containing starch and glycerol (Azumi et al., 2008). In this study, to normalize the growth condition for comparative analysis, the fungi were grown in a nutrient-rich SDB medium. It is thus possible that certain compound(s) will not be produced by the fungi grown in this condition. Chemical diversity analysis is still required by growing the fungi in different conditions, especially that the insect-host-oriented stimulations may facilitate the identification of novel compounds from these fungi.

As indicated above, the putative gene cluster for helvolic acid biosynthesis is not present in the specialist fungi (Donzelli and Krasnoff, 2016). However, our metabolomic analysis detected the compound in *M. acridum* and *M. album* (**Figure 5A**; **Supplementary Table S2**). The biosynthetic mechanism of helvolic acid has not been fully understood. A loss-of-function study revealed that a geranylgeranyl diphosphate synthase (BAJ05823) is involved in the production of helvolic acid in *M. anisopliae* (Singkaravanit et al., 2010). Our genome survey indicated that the homolog of this protein is present not only in the non-specialists (e.g., MAA_03020 of *M. robertsii*, 82% identity) but also in *M. acridum* (MAC_02619, 80%) and *M. album* (MAM_05601, 75%). A previous study in *Aspergillus fumigatus* indicated that an oxidosqualene cyclase (Afu4g14770) is required for helvolic acid biosynthesis (Mitsuguchi et al., 2009). Likewise, the homolog of this cyclase is present in *M. robertsii* (MAA_06578, 63%), *M. acridum* (MAC_09947, 38%), and *M. album* (MAM_07210, 39%). However, the homologs of acyltransferases (Afu4g14820 and Afu4g14820) are not present in the genomes of the specialist fungi. Thus, the biosynthetic nature of helvolic acid in *Metarhizium* species remains to be determined in the future.

## Conclusion

We performed comprehensive genomics and metabolomics analyses of seven insect pathogenic *Metarhizium* species by focusing on their gene contents and abilities in producing bioactive metabolites. The gene clusters involved in the biosynthesis of secondary metabolites are diverse among the examined species. The presence of non-selective insecticidal toxin producing gene cluster in non-specialist species is generally associated with fungal adaptation to diverse insect hosts. Consistent with the biosynthetic gene diversification, the MS-based metabolomics data also revealed the chemical diversification among the seven *Metarhizium* species, which could separate the fungal species into groups correlating with fungal host ranges. The results obtained in this study will benefit future investigations and elucidations of secondary metabolic mechanisms and the function of small molecules involved in insect-fungal interactions.

## Author Contributions

Y-JX, and FL performed experiments. Y-JX, BL, YS, and CW analyzed the data. Y-JX and CW wrote the manuscript. CW designed the experiments.

## Conflict of Interest Statement

The authors declare that the research was conducted in the absence of any commercial or financial relationships that could be construed as a potential conflict of interest.
