# Supplementary material for: Metabolic Conservation and Diversification of Metarhizium Species Correlate with Fungal Host-Specificity
Source: Front Microbiol. 2016 Dec 16;7:2020. doi: 10.3389/fmicb.2016.02020 (PMC5159617; doi:10.3389/fmicb.2016.02020)
Supplement: Supplementary file 1 [file Table_1.DOCX]

**Table S1. The metabolites identified in hypha in *Metarhizium* species using GC-MS.**

| **m/z** | **RT** | **Metabolite** | **The mean peak area in each *Metarhizium* species** | | | | | | |
| --- | --- | --- | --- | --- | --- | --- | --- | --- | --- |
|  |  |  | **MAA** | **MAM** | **MGU** | **MAJ** | **MAN** | **MBR** | **MAC** |
| 87.02 | 3.36 | Glycerate | 3.98E+06 | 3.98E+06 | 3.98E+06 | 3.98E+06 | 4.71E+08 | 1.52E+08 | 1.26E+08 |
| 71.99 | 5.15 | Oxalate | 3.98E+06 | 3.98E+06 | 3.98E+06 | 3.98E+06 | 1.10E+09 | 3.98E+06 | 3.98E+06 |
| 42.02 | 5.80 | Valine | 3.98E+06 | 3.98E+06 | 2.30E+08 | 1.06E+09 | 4.73E+08 | 6.55E+08 | 8.89E+08 |
| 102.03 | 6.45 | Leucine | 1.51E+09 | 3.98E+06 | 1.84E+09 | 4.89E+09 | 3.98E+06 | 2.38E+09 | 4.12E+09 |
| 240.01 | 6.87 | Serine | 3.98E+06 | 3.98E+06 | 3.98E+06 | 3.98E+06 | 3.98E+06 | 6.32E+08 | 6.13E+08 |
| 227.99 | 7.33 | Tyrosine | 1.08E+09 | 1.60E+09 | 1.46E+09 | 3.15E+09 | 2.25E+09 | 2.18E+09 | 1.92E+09 |
| 237.04 | 7.48 | Galactose | 1.70E+07 | 3.98E+06 | 3.98E+06 | 3.98E+06 | 4.88E+08 | 4.28E+08 | 3.98E+06 |
| 115.02 | 7.56 | Ornithine | 1.37E+09 | 9.55E+08 | 1.14E+09 | 3.98E+06 | 1.51E+09 | 1.04E+09 | 3.98E+06 |
| 114.04 | 7.57 | Acetyl-Lysine | 3.98E+06 | 3.98E+06 | 3.98E+06 | 3.89E+07 | 9.59E+08 | 1.85E+08 | 3.98E+06 |
| 147.00 | 7.65 | Malonate | 4.21E+09 | 1.97E+10 | 2.64E+09 | 1.74E+10 | 1.35E+10 | 2.33E+10 | 2.24E+10 |
| 294.05 | 7.90 | Asparate | 3.98E+06 | 2.41E+08 | 3.98E+06 | 3.02E+08 | 3.98E+06 | 3.98E+06 | 4.76E+08 |
| 233.03 | 7.95 | Erythritol | 8.52E+08 | 1.08E+09 | 5.97E+08 | 1.29E+09 | 8.58E+08 | 1.17E+09 | 3.55E+08 |
| 140.02 | 8.00 | Proline | 7.83E+08 | 8.26E+08 | 5.82E+08 | 1.45E+09 | 8.99E+08 | 1.40E+09 | 1.09E+09 |
| 56.03 | 8.54 | Glutamate | 2.26E+08 | 4.25E+08 | 1.72E+08 | 2.87E+08 | 5.93E+08 | 1.40E+09 | 1.23E+08 |
| 91.01 | 8.63 | Phenylalanine | 3.98E+06 | 9.01E+08 | 3.98E+06 | 6.29E+08 | 3.98E+06 | 3.98E+06 | 3.98E+06 |
| 309.05 | 9.19 | Xylitol | 3.98E+06 | 3.98E+06 | 3.98E+06 | 3.98E+06 | 6.04E+08 | 3.98E+06 | 3.98E+06 |
| 185.03 | 9.66 | Galactitol | 1.19E+09 | 3.98E+06 | 2.35E+08 | 3.98E+06 | 1.68E+09 | 1.08E+09 | 3.98E+06 |
| 183.00 | 9.71 | Citrate | 8.11E+08 | 1.33E+08 | 8.77E+08 | 7.59E+08 | 3.63E+08 | 5.43E+08 | 3.98E+06 |
| 170.04 | 10.01 | Glucose | 3.98E+06 | 1.19E+09 | 3.98E+06 | 7.10E+08 | 2.25E+08 | 1.42E+08 | 3.98E+06 |
| 264.98 | 10.19 | Lysine | 3.98E+06 | 6.18E+08 | 3.98E+06 | 3.98E+06 | 3.98E+06 | 3.98E+06 | 3.98E+06 |
| 238.04 | 10.24 | Histidine | 3.98E+06 | 3.98E+06 | 3.98E+06 | 7.11E+08 | 4.72E+08 | 4.63E+08 | 2.38E+08 |
| 154.01 | 10.26 | Sorbitol | 3.98E+06 | 8.49E+08 | 3.98E+06 | 3.98E+06 | 3.98E+06 | 3.98E+06 | 3.98E+06 |
| 193.02 | 10.53 | Glucosamine | 8.95E+08 | 3.98E+06 | 3.98E+06 | 2.27E+08 | 1.54E+08 | 2.77E+08 | 3.98E+06 |
| 437.05 | 10.59 | Mannitol | 3.98E+06 | 4.86E+08 | 3.98E+06 | 3.98E+06 | 3.98E+06 | 3.98E+06 | 3.98E+06 |
| 132.02 | 10.75 | Octadecadienoic acid | 1.59E+09 | 3.70E+08 | 7.30E+08 | 3.11E+09 | 1.97E+09 | 1.04E+09 | 9.68E+08 |
| 201.02 | 10.77 | Glycerate-3P | 3.98E+06 | 7.86E+08 | 3.98E+06 | 3.98E+06 | 3.98E+06 | 3.98E+06 | 3.98E+06 |
| 53.00 | 11.53 | Octadecadienoic acid | 3.98E+06 | 2.13E+08 | 3.98E+06 | 1.11E+09 | 8.64E+08 | 6.22E+08 | 1.04E+08 |
| 80.03 | 11.54 | Linoleic acid | 4.08E+08 | 4.78E+08 | 2.99E+08 | 5.95E+08 | 7.81E+08 | 6.83E+08 | 5.38E+08 |
| 278.00 | 11.60 | Stearic acid | 3.98E+06 | 8.90E+08 | 3.98E+06 | 3.98E+06 | 3.98E+06 | 3.98E+06 | 3.98E+06 |
| 203.01 | 11.68 | Tryptophan | 2.80E+08 | 7.67E+08 | 1.28E+08 | 1.24E+09 | 2.16E+08 | 2.47E+08 | 1.09E+08 |
| 198.03 | 12.40 | Oleamide | 2.13E+08 | 3.98E+06 | 1.82E+08 | 7.40E+08 | 8.56E+08 | 7.77E+08 | 1.91E+08 |
| 298.04 | 13.14 | Aucubin | 3.98E+06 | 3.11E+08 | 3.98E+06 | 1.31E+08 | 3.98E+06 | 8.19E+07 | 3.98E+06 |
| 237.05 | 13.39 | Adenosine | 2.48E+08 | 2.91E+08 | 2.99E+08 | 8.83E+08 | 3.98E+06 | 3.98E+06 | 3.98E+06 |
| 363.07 | 14.05 | Trehalose | 1.25E+08 | 1.17E+09 | 1.76E+08 | 5.85E+08 | 3.98E+06 | 1.10E+09 | 7.55E+08 |
| 378.20 | 17.52 | Sitosterol | 3.98E+06 | 3.98E+06 | 3.98E+06 | 6.91E+07 | 4.15E+07 | 4.29E+07 | 3.98E+06 |
