# Supplementary material for: Metabolic Conservation and Diversification of Metarhizium Species Correlate with Fungal Host-Specificity
Source: Front Microbiol. 2016 Dec 16;7:2020. doi: 10.3389/fmicb.2016.02020 (PMC5159617; doi:10.3389/fmicb.2016.02020)
Supplement: Supplementary file 2 [file Table_2.DOCX]

**Table S2. The metabolites identified in hypha in *Metarhizium* species using LC-MS.**

| **m/z** | **RT**  **(min)** | **Metabolites*** | **The mean peak area in each *Metarhizium* species** | | | | | | |
| --- | --- | --- | --- | --- | --- | --- | --- | --- | --- |
|  |  |  | **MAM** | **MAC** | **MAJ** | **MGU** | **MBR** | **MAA** | **MAN** |
| 566.33 | 27.75 | Proto Dtx B | ND | ND | ND | 2.13E+02 | 7.75E+01 | 1.55E+02 | 2.28E+02 |
| 610.34 | 21.49 | hydroxy-Dtx B | ND | ND | ND | 5.75E+04 | 6.60E+03 | 1.94E+04 | 7.31E+03 |
| 608.36 | 25.55 | Homo-Dtx B | ND | ND | ND | ND | 4.50E+02 | 7.29E+02 | 3.81E+02 |
| 596.36 | 21.35 | Dtx F | ND | ND | ND | 1.72E+04 | 3.02E+03 | 7.16E+03 | 3.19E+03 |
| 580.37 | 29.13 | Dtx E2 | ND | ND | ND | 7.29E+03 | ND | ND | 1.43E+03 |
| 616.37 | 29.37 | Dtx E1 Diol | ND | ND | ND | 1.79E+05 | 9.23E+04 | 1.10E+05 | 9.79E+04 |
| 608.40 | 31.07 | Dtx E1 | ND | ND | ND | 4.51E+03 | 3.07E+03 | 2.95E+03 | 2.76E+03 |
| 630.33 | 23.43 | Dtx E Chlorohydrin | ND | ND | ND | 2.38E+05 | 8.00E+04 | 1.59E+05 | 5.65E+04 |
| 594.39 | 29.36 | Dtx E | ND | ND | ND | 2.67E+05 | 1.19E+05 | 1.75E+05 | 1.31E+05 |
| 624.36 | 22.83 | Dtx D2 | ND | ND | ND | 2.23E+05 | 7.05E+04 | 1.30E+05 | 7.05E+04 |
| 638.29 | 22.06 | Dtx D1 | ND | ND | ND | 3.84E+04 | 8.67E+03 | 1.41E+04 | 6.14E+03 |
| 624.36 | 22.34 | Dtx D | ND | ND | ND | 2.30E+04 | 6.15E+03 | 1.78E+04 | 6.91E+03 |
| 582.37 | 27.30 | Dtx C2 | ND | ND | ND | 4.77E+03 | 1.41E+03 | 2.25E+03 | 7.65E+02 |
| 610.38 | 22.73 | Dtx C | ND | ND | ND | 3.14E+04 | 1.12E+04 | 2.30E+04 | 1.00E+04 |
| 580.37 | 27.30 | Dtx B2 | ND | ND | ND | 9.44E+04 | 3.01E+04 | 4.53E+04 | 1.72E+04 |
| 608.36 | 24.47 | Dtx B1 | ND | ND | ND | 3.01E+04 | 6.61E+03 | 1.83E+04 | 4.11E+03 |
| 606.32 | 26.22 | Dtx A5 | ND | ND | ND | 4.01E+02 | 1.72E+02 | 3.53E+02 | ND |
| 644.35 | 25.70 | Dtx A4 Chrorohydrin | ND | ND | ND | ND | 5.80E+02 | 7.54E+02 | 7.47E+02 |
| 592.37 | 27.94 | Dtx A4 | ND | ND | ND | 2.18E+03 | 6.34E+02 | 1.43E+03 | 7.86E+02 |
| 566.35 | 25.90 | Dtx A3 | ND | ND | ND | 1.11E+04 | 1.83E+03 | 1.57E+03 | 9.68E+02 |
| 564.34 | 24.26 | Dtx A2 | ND | ND | ND | 9.43E+03 | 1.48E+03 | 5.00E+03 | 2.02E+03 |
| 592.37 | 29.00 | Dtx A1 | ND | ND | ND | 2.46E+02 | 2.90E+03 | 1.86E+03 | 2.41E+03 |
| 578.35 | 26.27 | Dtx A | ND | ND | ND | 3.65E+05 | 1.61E+05 | 2.17E+05 | 1.26E+05 |
| 580.36 | 26.26 | Dihydroxy-Dtx A | ND | ND | ND | 4.02E+04 | 1.22E+04 | 2.01E+04 | 8.91E+03 |
| 566.35 | 24.78 | Desmethyl-Dtx B2 | ND | ND | ND | 1.08E+04 | 1.44E+03 | 3.02E+03 | 1.11E+03 |
| 580.37 | 27.93 | Desmethyl-Dtx B | ND | ND | ND | 1.62E+05 | 3.47E+04 | 8.42E+04 | 4.05E+04 |
| 564.34 | 24.78 | Desmethyl-Dtx A | ND | ND | ND | 1.73E+05 | 2.83E+04 | 5.54E+04 | 2.20E+04 |
| 439.29 | 12.83 | Debromomethoxymarinone | 1.40E+03 | ND | ND | 1.44E+02 | 5.23E+02 | ND | 9.64E+01 |
| 477.14 | 17.36 | 1,3,6,8-Tetrahydroxy-2-methylanthraquinone | ND | ND | ND | 1.43E+03 | ND | ND | ND |
| 332.13 | 9.26 | 1,4-Dihydroxy-2-(2-pyridylmethyl)anthraquinone | ND | ND | 2.60E+02 | 2.33E+03 | 1.30E+03 | 1.19E+03 | 7.93E+02 |
| 330.09 | 4.11 | 1-Bromoanthraquinone-2-carboxylic acid | ND | ND | 4.88E+02 | 7.46E+01 | ND | 2.50E+02 | 2.34E+02 |
| 457.11 | 18.83 | 1-Hydroxysulfurmycin A | ND | ND | ND | 2.73E+03 | ND | ND | ND |
| 223.07 | 6.94 | 1-Methylanthraquinone | ND | ND | ND | 4.70E+02 | ND | ND | ND |
| 289.14 | 11.87 | 2-(4-Methyl-1,3-pentadienyl)anthraquinone | ND | ND | ND | 3.75E+03 | 9.81E+02 | 2.41E+03 | 2.09E+03 |
| 375.14 | 12.90 | 2,8-Dihydroxy-6-hydroxymethyl-1,3,5-trimethoxyanthraquinone | ND | ND | 1.38E+02 | 1.37E+03 | 1.04E+03 | 1.25E+03 | 8.83E+02 |
| 301.07 | 16.15 | 2-Methoxy-1-nitroanthraquinone | ND | ND | ND | 1.81E+03 | ND | ND | ND |
| 223.08 | 7.39 | 2-Methylanthraquinone | ND | ND | 5.54E+01 | 9.24E+02 | ND | 2.41E+02 | 6.82E+01 |
| 355.05 | 3.05 | 3',4'-Dehydro-4'-deoxydothistromin | 3.96E+02 | 1.02E+02 | ND | 1.39E+03 | 1.79E+02 | 1.03E+03 | 3.84E+02 |
| 289.14 | 11.08 | 4-Hydroxy-9-methoxy--lapachone | ND | ND | ND | 5.58E+02 | ND | 2.24E+02 | 1.42E+02 |
| 209.10 | 3.19 | 9,10-Anthraquinon | 5.39E+01 | 9.80E+01 | 1.15E+02 | 3.19E+02 | 1.00E+02 | 1.90E+02 | 1.78E+02 |
| 413.13 | 2.73 | Aklavinone | ND | ND | ND | ND | ND | 1.73E+02 | ND |
| 387.17 | 2.72 | Annulin B | 1.00E+02 | ND | ND | 8.72E+01 | ND | 1.93E+02 | ND |
| 436.11 | 2.76 | Anthracyclinone blue A | 3.04E+01 | ND | ND | 3.28E+02 | ND | 4.81E+01 | ND |
| 309.16 | 2.54 | Anthrakunthone | 1.38E+03 | 8.69E+01 | 9.76E+01 | 8.16E+02 | 2.82E+02 | 3.08E+02 | 7.23E+02 |
| 259.06 | 17.36 | Anthraquinone-1,8-dicarboxylic acid | ND | ND | ND | 4.82E+02 | ND | ND | ND |
| 691.06 | 17.44 | Antibiotic IB 00208 | 1.26E+02 | ND | ND | ND | ND | ND | ND |
| 349.13 | 10.09 | Antibiotic M 13-1 | 8.07E+02 | ND | 1.20E+02 | ND | ND | ND | 5.66E+01 |
| 437.15 | 12.39 | Antibiotic UCE 6 | 1.59E+02 | ND | ND | ND | ND | ND | ND |
| 327.05 | 2.81 | Araliolactone A | 1.35E+03 | 1.61E+03 | 7.93E+02 | 3.39E+03 | 1.79E+03 | 1.70E+03 | 1.00E+02 |
| 307.15 | 11.30 | Bungone A | ND | ND | ND | 2.74E+03 | 8.52E+02 | 1.72E+03 | 2.00E+03 |
| 337.18 | 3.24 | Bungone B | 5.74E+02 | ND | ND | ND | 7.08E+01 | ND | 1.47E+02 |
| 380.12 | 3.46 | C.I. Disperse Blue 60 | 1.66E+02 | ND | ND | ND | ND | ND | ND |
| 355.19 | 5.79 | Citromycinone | 1.96E+02 | 1.21E+02 | ND | 3.34E+02 | 9.95E+01 | 5.36E+01 | 1.52E+02 |
| 283.06 | 4.01 | Damnacanthal | ND | 3.16E+01 | 2.18E+02 | ND | ND | 2.02E+02 | 1.17E+02 |
| 425.19 | 9.16 | Debromohydroxymarinone | ND | 5.72E+01 | 1.34E+02 | ND | ND | 2.03E+02 | 2.00E+02 |
| 367.12 | 3.56 | HMP-M2 | 1.82E+02 | 1.79E+02 | ND | ND | ND | 2.41E+02 | ND |
| 259.10 | 7.09 | Hydroxy Lapachone | 1.89E+02 | 2.90E+02 | 4.95E+02 | 9.66E+02 | 2.29E+02 | 3.56E+02 | 2.86E+01 |
| 273.15 | 10.49 | methoxy Lapachone | 2.60E+02 | ND | ND | ND | ND | ND | ND |
| 355.18 | 4.12 | Naphterpin | 6.78E+02 | 2.93E+02 | ND | 1.38E+03 | 5.07E+02 | ND | ND |
| 513.23 | 12.18 | Naphthablin | 9.44E+02 | 1.70E+02 | 6.02E+01 | 6.68E+02 | 6.89E+02 | 8.73E+02 | 7.59E+01 |
| 357.19 | 4.11 | Naphthgeranine B | 2.80E+02 | ND | ND | 2.64E+02 | 2.80E+02 | ND | ND |
| 373.19 | 4.12 | Naphthgeranine C | 1.00E+02 | ND | ND | 4.22E+02 | 1.75E+02 | 1.69E+02 | 2.57E+02 |
| 389.18 | 3.70 | Naphthgeranine D | 8.59E+02 | ND | ND | ND | 2.56E+02 | 4.50E+02 | 7.59E+02 |
| 353.13 | 2.66 | Vismiaquinone | 3.73E+02 | ND | ND | 2.63E+02 | 1.32E+02 | 1.59E+02 | 2.72E+02 |
| 216.14 | 11.95 | 1-Methoxy-3,5-dinitro-2(3H)-pyridinone | 8.90E+01 | ND | ND | ND | ND | ND | ND |
| 296.12 | 8.45 | Akanthomycin | ND | ND | ND | ND | ND | 4.10E+02 | 2.09E+03 |
| 422.23 | 4.07 | Antibiotic TMC 72 | 4.30E+02 | 3.69E+02 | 4.52E+02 | 1.52E+03 | 2.89E+02 | 1.04E+03 | ND |
| 446.22 | 7.95 | Apiosporamide | ND | 1.75E+02 | ND | 7.86E+02 | 2.27E+02 | 2.47E+02 | ND |
| 208.07 | 7.29 | Ciclopirox | ND | ND | ND | ND | ND | ND | 1.79E+02 |
| 292.11 | 8.66 | Cordypyridones D | 9.27E+02 | ND | 1.98E+02 | 2.98E+02 | 1.31E+02 | ND | 6.22E+01 |
| 508.24 | 14.27 | Cytochalasin Q | ND | 6.15E+02 | 1.36E+02 | 4.80E+02 | ND | ND | ND |
| 432.13 | 14.93 | Fischerin | ND | ND | 4.87E+02 | ND | ND | ND | ND |
| 292.10 | 3.54 | Fusaricide | 1.61E+03 | ND | 3.35E+02 | 8.60E+02 | 4.11E+02 | ND | ND |
| 462.24 | 11.56 | GKK1032A2 | 1.30E+03 | ND | ND | ND | ND | ND | ND |
| 206.11 | 13.78 | Haplotusine | ND | ND | ND | ND | 8.30E+03 | 1.26E+03 | 3.59E+03 |
| 446.23 | 8.39 | Hirsutellone A | 4.02E+01 | 2.28E+02 | 6.19E+01 | 5.30E+02 | 4.71E+02 | 5.35E+02 | 4.77E+01 |
| 448.22 | 8.99 | Hirsutellone B | 7.80E+02 | 1.24E+03 | 9.90E+02 | 2.95E+03 | 1.52E+03 | 2.50E+03 | 1.20E+03 |
| 462.20 | 4.18 | Hirsutellone C | 4.55E+02 | 3.19E+02 | 5.87E+01 | 7.17E+02 | 4.13E+02 | 5.61E+02 | 2.01E+02 |
| 140.07 | 3.51 | Metipirox | 2.98E+02 | 7.39E+02 | 2.87E+02 | 6.08E+01 | 9.45E+02 | 1.57E+03 | 2.38E+03 |
| 460.20 | 5.98 | Militarinone A | ND | ND | ND | 5.56E+01 | ND | 1.87E+02 | ND |
| 582.30 | 7.77 | Pyridomacrolidin | 5.70E+03 | 2.17E+02 | 2.03E+02 | 7.37E+02 | 3.08E+02 | 2.22E+02 | 2.17E+02 |
| 386.24 | 10.49 | Pyridoverich | 2.89E+03 | ND | ND | ND | 8.50E+01 | ND | ND |
| 264.12 | 8.63 | Pyridoxatin | 8.61E+02 | 1.60E+02 | 2.91E+02 | 2.40E+02 | 8.90E+01 | 6.80E+01 | 9.65E+01 |
| 358.15 | 19.74 | Rilopirox | ND | ND | 4.74E+02 | ND | 1.03E+02 | ND | 1.12E+02 |
| 444.24 | 8.30 | Torrubiellutin A | 2.74E+03 | ND | ND | 2.56E+03 | 1.80E+03 | 2.34E+02 | ND |
| 486.25 | 4.06 | Torrubiellutin B | 1.33E+02 | 1.84E+02 | ND | 3.46E+02 | 4.57E+01 | ND | ND |
| 528.26 | 5.84 | Torrubiellutin C | 7.71E+02 | 2.91E+02 | 1.96E+02 | 1.22E+03 | 5.83E+02 | 6.74E+02 | 5.13E+02 |
| 337.16 | 19.66 | 5-Chloro, 5'-oxo-Colletorin B | ND | ND | ND | ND | 2.93E+02 | ND | 5.51E+02 |
| 355.19 | 5.79 | 6'-Ketone-Colletochlorin A | 1.96E+02 | 1.21E+02 | ND | 3.34E+02 | 9.95E+01 | 5.36E+01 | 1.52E+02 |
| 403.18 | 6.27 | 8',9'-Didehydro-Ascochlorin | ND | 4.93E+02 | ND | ND | ND | ND | ND |
| 325.23 | 39.87 | Acetoxyscirpenediol | 5.31E+01 | 9.51E+01 | ND | ND | 3.72E+01 | ND | 1.01E+02 |
| 405.18 | 4.10 | Ascochlorin | ND | 4.18E+01 | ND | 4.19E+02 | 2.39E+02 | 9.74E+01 | ND |
| 423.19 | 6.09 | Ascofuranol | ND | ND | 2.87E+02 | 1.02E+03 | 8.90E+02 | 1.62E+03 | 1.21E+03 |
| 245.13 | 12.68 | Colletochlorin D | ND | ND | ND | 7.80E+01 | 2.71E+02 | 4.94E+02 | ND |
| 323.13 | 34.50 | Colletorin B | 1.08E+02 | 6.80E+01 | 7.74E+01 | 2.24E+02 | 8.06E+01 | 6.55E+01 | 1.45E+02 |
| 407.16 | 9.14 | Grifolic acid | ND | 1.42E+02 | 3.57E+01 | 2.59E+02 | ND | 1.82E+02 | 7.90E+01 |
| 569.30 | 9.71 | Helvolic acid | 2.17E+02 | 1.92E+03 | 1.06E+03 | 2.31E+03 | 1.27E+03 | 2.90E+03 | 5.29E+02 |
| 283.06 | 4.01 | Paecilomycine B | ND | 3.16E+01 | 2.18E+02 | ND | ND | 2.02E+02 | 1.17E+02 |
| 265.11 | 13.66 | Paecilomycine C | ND | 4.99E+02 | 1.66E+02 | 6.13E+02 | 1.82E+02 | 3.19E+02 | ND |
| 267.11 | 3.40 | Spirotenuipesine A | 6.22E+02 | 9.55E+02 | 6.23E+02 | 2.37E+03 | 8.46E+02 | 1.34E+03 | 4.09E+02 |
| 283.15 | 7.03 | Spirotenuipesine B | ND | ND | ND | ND | ND | 4.03E+02 | ND |
| 325.23 | 11.88 | Tenuipesine A | ND | ND | ND | 8.58E+03 | 2.23E+03 | 4.94E+03 | 4.88E+03 |

*Note: Dtx, destruxin; ND, not detected.
